# Grief and its impact in older adults: Findings from the National Social Life, Health, and Aging Project, Wave 2

**DOI:** 10.1093/geroni/igaf122.3948

**Published:** 2025-12-31

**Authors:** Swasati Handique

**Affiliations:** University of Texas at Arlington, Arlington, Texas, United States

## Abstract

This study examined how feelings of being stunned and angry after the death of someone close have an impact on the physical and mental health of older adults. Data for this study was drawn from a nationally representative sample of 3196 individuals (aged 60-plus) from the NSHAP, Wave 2. The primary outcomes were self-rated physical health and mental health, measured on a 5-point Likert scale, with independent variables as responses to death of feeling stunned and feeling angry, both measured on a 5-point scale. Multiple linear regression analyses was conducted using Stata 17.0.

**Results:**

Descriptives revealed a mean age of 73.2 years, with the sample being 53% female. Self-rated physical health was statistically significant, F(6, 1935) = 18.05, p < .001. Feeling stunned was negatively associated with physical health (β = -0.10, p < .001), suggesting that those who reported being more emotionally overwhelmed by a death also rated their physical health lower. Self-rated mental health was also statistically significant, F(6, 1938) = 20.11, p < .001. Feeling stunned (β = -0.10, p < .001) and feeling angry (β = -0.11, p < .001) were significant negative predictors of mental health. Findings from this study highlight that prolonging grief has the potential of awakening the memories of past losses compounding the grief and impacting the physical and mental health of older adults. Therefore, grief support and community-based groups can be serve as essential resources to foster grief literacy and provide support to older adults navigating a loss.

